# The complete sequence of the mitochondrial genome of *Nautilus macromphalus *(Mollusca: Cephalopoda)

**DOI:** 10.1186/1471-2164-7-182

**Published:** 2006-07-19

**Authors:** Jeffrey L Boore

**Affiliations:** 1 Evolutionary Genomics Program, DOE Joint Genome Institute and Lawrence Berkeley National Laboratory, Walnut Creek, CA, 94598, USA; 2 Department of Integrative Biology, University of California, Berkeley, CA, USA 94720 and Genome Project Solutions, Hercules, CA, 94547, USA

## Abstract

**Background:**

Mitochondria contain small genomes that are physically separate from those of nuclei. Their comparison serves as a model system for understanding the processes of genome evolution. Although complete mitochondrial genome sequences have been reported for more than 600 animals, the taxonomic sampling is highly biased toward vertebrates and arthropods, leaving much of the diversity yet uncharacterized.

**Results:**

The mitochondrial genome of the bellybutton nautilus, *Nautilus macromphalus*, a cephalopod mollusk, is 16,258 nts in length and 59.5% A+T, both values that are typical of animal mitochondrial genomes. It contains the 37 genes that are almost universally found in animal mtDNAs, with 15 on one DNA strand and 22 on the other. The arrangement of these genes can be derived from that of the distantly related *Katharina tunicata *(Mollusca: Polyplacophora) by a switch in position of two large blocks of genes and transpositions of four tRNA genes. There is strong skew in the distribution of nucleotides between the two strands, and analysis of this yields insight into modes of transcription and replication. There is an unusual number of non-coding regions and their function, if any, is not known; however, several of these demark abrupt shifts in nucleotide skew, and there are several identical sequence elements at these junctions, suggesting that they may play roles in transcription and/or replication. One of the non-coding regions contains multiple repeats of a tRNA-like sequence. Some of the tRNA genes appear to overlap on the same strand, but this could be resolved if the polycistron were cleaved at the beginning of the downstream gene, followed by polyadenylation of the product of the upstream gene to form a fully paired structure.

**Conclusion:**

*Nautilus macromphalus *mtDNA contains an expected gene content that has experienced few rearrangements since the evolutionary split between cephalopods and polyplacophorans. It contains an unusual number of non-coding regions, especially considering that these otherwise often are generated by the same processes that produce gene rearrangements. The skew in nucleotide composition between the two strands is strong and associated with the direction of transcription in various parts of the genomes, but a comparison with *K. tunicata *implies that mutational bias during replication also plays a role. This appears to be yet another case where polyadenylation of mitochondrial tRNAs restores what would otherwise be an incomplete structure.

## Background

Animal mitochondrial DNA (mtDNA) is nearly always a closed circular molecule and, with a few exceptions [e.g. [1-4]], contains the same 37 genes, specifying 13 proteins, two ribosomal RNAs, and 22 tRNAs [5]. Sequences of these diminutive genomes have been broadly used to address phylogenetic questions ranging from the population [6,7] to the interphylum [8-11] levels and to model many processes of genome evolution [12,13]. Although there are exceptions, most mtDNAs contain no introns and are between 14 and 17 kb. Typically there are few intergenic nucleotides except for a single large non-coding region generally thought to contain elements that regulate replication and transcription [14]. Occasionally non-coding regions have been found that contain repeated elements [15] or contain pseudogenes [12,16] or that may be remnants of duplicated regions, perhaps those that mediate gene rearrangements [12,16,17]. Gene rearrangements tend to be uncommon and to occur in a saltatory manner [see [10]]. The "universal" genetic code has been modified in many animal lineages, to include the use of alternative start codons and abbreviated stop codons [18,19]. In some mtDNAs there is pronounced skew in nucleotide composition, often with one strand being rich in G and T and the other in A and C [20]. Post-transcriptional modification of nucleotides has been observed for tRNAs [21,22].

Little study has been done to date on mollusk mtDNAs compared to those of vertebrates or arthropods [23], but it is already apparent that mollusks exhibit much variation in the features of their mitochondrial genomes, including losses and gains of genes [2], atypically large amounts of duplicated or non-coding nucleotides [15,24], highly rearranged genomes [2,25], and an unusual pattern of passage termed doubly uniparental inheritance [26,27]. This is furthered here by reporting and comparing the features of the mitochondrial genome of the first nautiloid to be so studied, *N. macromphalus *(Mollusca: Cephalopoda).

Nautiloids were once abundant and diverse in the Paleozoic seas, but only a handful of species remain. They are part of the molluscan class Cephalopoda, which otherwise contains octopi, squid, and cuttlefish. They are the earliest diverging lineage of this group and are often considered to be "living fossils" since living forms seem to have changed little from their ancient ancestors. They live in spiral-shaped shells which are filled with gas to control buoyancy and they move about by squirting jets of water. They are carnivorous, using their many grooved tentacles to grasp prey and pass it to their mouth, where a beak-like jaw tears it and passes it to the shredding radula. They live throughout the Southwest Pacific Ocean, at depths as great as 610 meters, and traverse a great range, as shallow as 90 meters, apparently in search of prey.

Complete mtDNA sequences have been determined for 23 mollusks (see Additional file 1), including a representative (*Katharina tunicata*) [28] of a basal group (Polyplacophora). This sampling includes seven other cephalopods: *Octopus vulgaris *[29], *Loligo bleekeri *[30], *Todarodes pacificus *[29,31], *O. ocellatus*, *Sepioteuthis lessoniana*, *Watasenia scintillans*, and *Sepia officinalis *[31]. Comparisons of the features of the *N. macromphalus *mtDNA with those of some other mollusks are presented here.

## Results and discussion

### Gene content and organization

**Figure 1:**
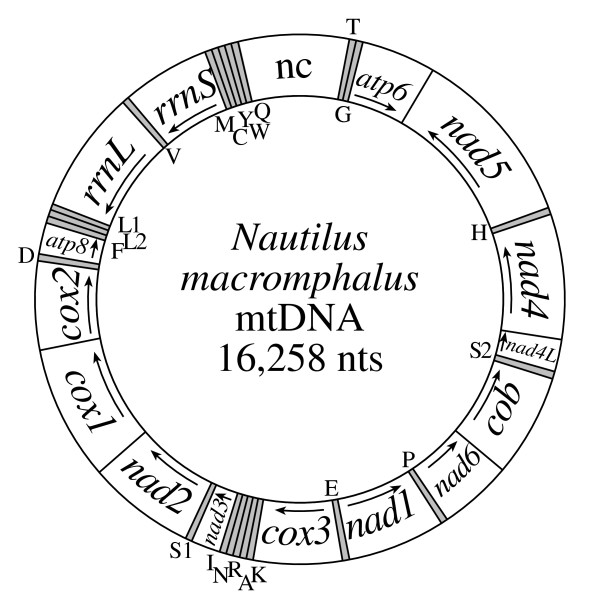
**Mitochondrial gene map of the cephalopod mollusk *Nautilus macromphalus***. Genes for proteins and rRNAs are shown with standard abbreviations with an arrow indicating the direction of transcription. Genes for tRNAs are designated by a single letter for the corresponding amino acid, with the two leucine and two serine tRNAs differentiated by numeral (S1, S2, L1, and L2 recognizing codons AGN, UCN, CUN, and UUR, respectively). tRNA genes shown outside the circle are transcribed clockwise and those inside the circle are transcribed counter-clockwise. The largest non-coding region is designated "nc".

Complete mtDNA sequences have been determined 23 mollusks. The *Nautilus macromphalus *(sometimes called the bellybutton nautilus) mitochondrial genome is 16,258 bp in length (GenBank accession number DQ472026) and contains the set of 37 genes most commonly found for animal mtDNAs [5]. Fifteen genes are located on one strand and 22 on the other (Fig. 1). There are several substantial non-coding regions (see below), the largest of which is 972 nts long and between *trnQ *and *trnT*.

**Figure 2:**
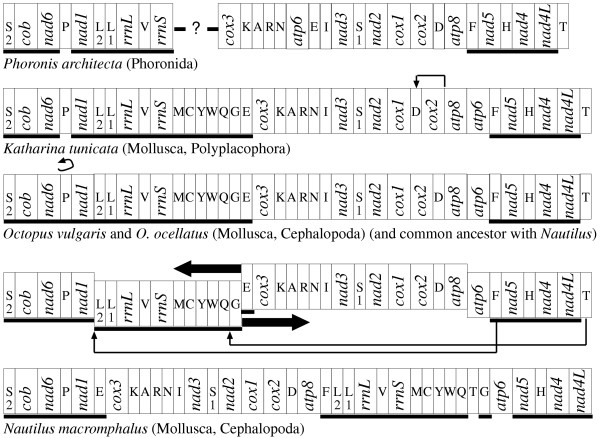
**Reconstruction of mitochondrial genome rearrangements for *Nautilus macromphalus***. At the top is the nearly complete gene arrangement for *Phoronis architecta *[11], a presumed outgroup to the mollusks, shown to polarize two of the cephalopod rearrangements: Having *trnP *in opposite orientation to *nad6 *and *nad1 *is the ancestral condition, as is having *trnD *between *cox2 *and *atp8. *The only two differences between the chiton *Katharina tunicata *[28] and the two octopus species is the inversion of *trnP *in the octopus and the transposition of *trnD *in the chiton. (No attempt is being made here to reconstruct all of the rearrangements between the phoronid and the chiton.) The arrangement found in the *N. macromphalus*, then, can be reconstructed by the additional switch in order of two large blocks of genes plus transpositions of *trnF *and *trnT*. Genes are not drawn to scale and are abbreviated as in Fig. 1 except that underlining signifies right-to-left transcriptional orientation. All genomes are circular and only graphically linearized at an arbitrarily chosen point. These genomes are chosen to illustrate the paucity of rearrangements in these particular lineages. The cuttlefish and several squid with complete mtDNA sequences (see text) have experienced many rearrangements unique to their lineages, and these patterns are reconstructed by Akasaki et al. [31].

The mitochondrial gene arrangement of the distantly related *Katharina tunicata *[28] (the only sampled representative of the Polyplacophora, an early diverging class of the Mollusca) differs from that of another studied cephalopod, *Octopus vulgaris *[29], by only the inversion of *trnP *and a transposition of *trnD*, and differs from that of *N. macromphalus *by these changes plus additional transpositions of *trnF *and *trnT *and the switch in position of two large blocks of genes (Fig. 2). Therefore, each of these lineages has experienced very few gene rearrangements over several hundreds of millions of years. In order to determine which of these differences were caused by changes in the lineage leading to Polyphacophora versus those leading to the cephalopods, it is useful to identify more distantly related animals that share one or the other arrangement; since it seems very unlikely that identical rearrangements would occur in different lineages, one can reasonably infer that any gene arrangement shared by this outgroup taxon with either the polyplacophoran or a cephalopod is the ancestral condition for the common ancestor of the latter two groups. In this regard, the mitochondrial gene arrangement of a distantly related animal, the phoronid *Phoronis architecta *[11], is very useful since it has little diverged since these groups separated. From this comparison (and confirmed by others not shown), we can see that one of these tRNAs, *trnD*, remains in the ancestral condition in these two cephalopods, with the transposition having occurred in the polyplacophoran, whereas all other changes are derived for the cephalopods from that order parsimoniously inferred to be basal for the Mollusca.

In total, there are now available complete mtDNA sequences from eight cephalopod species to compare. In addition to *N. macromphalus *and *O. vulgaris*, these are the squids *Loligo bleekeri *[30], *Todarodes pacificus *[29,31], *Watasenia scintillans *[31], and *Sepioteuthis lessoniana *[31], the octopus *O. ocellatus *[31], and the cuttlefish *Sepia officinalis *[31]. *O. ocellatus *shares an identical gene arrangement with *O. vulgaris*. Two of the squids, *L. bleekeri *and *S. lessoniana*, share a nearly identical gene arrangement (differing only by a transposition of one block of genes: *trnI*, -*rrnL*, -*trnV*, -*rrnS*, -*trnW *[minus symbol indicates opposite transcriptional orientation]). This gene arrangement, plus another separately rearranged in *S. officinalis*, are highly derived and each shares only a few blocks of colinearity with the more conserved gene order of *N. macromphalus *mtDNA. All of these cephalopod mtDNAs have the same gene content except for *W. scintillans *and *T. pacificus*, the two representatives of the group Oegopsida. These two mtDNAs have a nearly identical gene arrangement, differing only in the position of *trnM*, that is highly rearranged from those of other mollusks, and contain duplicated copies of *cox1*, *cox2*, *cox3*, *atp6*, *atp8*, and *trnD*, such that they contain genes for a total of 18 proteins, 2 rRNAs, and 23 tRNAs. In all of these studied cephalopod mtDNAs, all genes retain the same transcriptional orientation, that is, all rearrangements are transpositions and none are inversions. Akasaki et al. [31] provide a comprehensive and well reasoned review of this pattern of arrangements, including proposals for mechanism of rearrangement, the role of the many large, non-coding regions, and evidence for concerted evolution of duplicated genes.

### Gene initiation and termination

Mitochondrial genomes often use a variety of non-standard initiation codons [19], but *N. macromphalus *mtDNA has only one type of deviation; three genes (*nad3*, *nad4*, and *nad5*) start with GTG and all others use the standard ATG (Additional file 4). Seven genes have unambiguous termination codons, either TAG (*atp6*,*cox1*, *nad5*) or TAA (*atp8*, *cox3*, *nad1*, *nad2*). In four cases (*cox2*, *cob*, *nad3*, *nad4*) genes are probably abbreviated to a single T or to TA such that the excision of the adjacent, downstream tRNA from the polycistronic message leaves an mRNA that is polyadenylated to complete a TAA stop codon. However, in each of these cases, a complete stop codon is available if there is, alternatively, overlap of only one or two nucleotides with the downstream tRNA. Perhaps these act as a "backup" for cases where translation precedes message cleavage. The other two cases are more ambiguous. *nad4L *could have an abbreviated stop codon, but is inferred to overlap *nad4 *by seven nucleotides to the first legitimate stop codon, since overlap of this pair has been commonly observed for other mtDNAs, where they are thought to be translated as a bicistron. *nad6 *is inferred to overlap *cob *by eight nucleotides, perhaps suggesting that these are processed also as a bicistron, but could instead end on an abbreviated stop codon if there were some signal for message cleavage (i.e., other than a tRNA) that we do not recognize. Inferred in this way, all protein-encoding genes have lengths nearly identical to those of *K. tunicata *mtDNA (Additional file 2).

### Transfer RNAs

**Figure 3:**
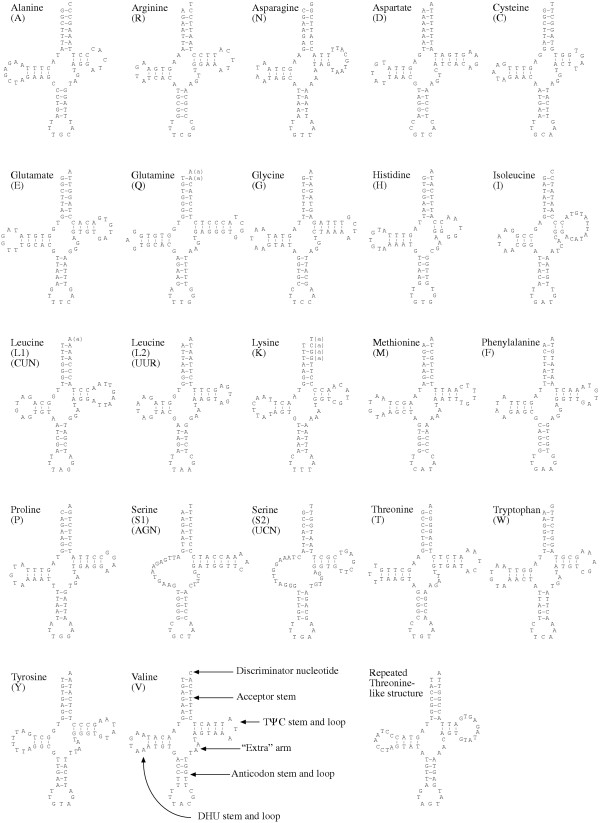
***Nautilus macromphalus *mitochondrial tRNA gene sequences folded into typical cloverleaf structures**. Lower case "a" in parentheses indicates likely replacements by (poly)adenylation after transcript cleavage at the downstream tRNA (see text for explanation). Structural features are shown on tRNA(V). Also shown is the secondary structure possible for the repeats in the large non-coding region that appear to be pseudogenes.

Sequences were identified whose potential secondary structures indicate that they encode the 22 tRNAs typically found for animal mtDNA (Fig. 3). In general, these appear well paired with only a few mismatches.

There are three cases where tRNA genes appear to overlap, and these potential structures suggest how this is resolved. *trnL1(nag) *appears to overlap *trnL2(yaa) *by only the former's discriminator nucleotide (A). *trnQ *appears to overlap *trnW *by two nucleotides. *trnK *appears to overlap *trnA *by four nucleotides, GGCT. These are well-paired in the potential structure of tRNA(A), but these four correspond to two G-T pairs, one mismatch, and the discriminator nucleotide of tRNA(K). It appears for each case that cleavage to form a complete downstream tRNA followed by (poly)adenylation of the upstream tRNA (as has been demonstrated for some mitochondrial tRNAs [22]) would yield fully formed, well-paired structures for all. This is illustrated in Figure 3 by lower case, parenthetical letter "a" appended to the genome-encoded nucleotide to indicate likely nucleotides in the actual transcript.

Usually T is in the first anticodon position for tRNAs that recognize either four-fold degenerate codon families or to specifically recognize NNR codons; G is usually in this position only to specifically recognize NNY codons. (Due to the convention of always drawing RNAs from 5' to 3' in orientation, the first nucleotide listed for an anticodon pairs with the last nucleotide of a codon.) All but two of the *N. macromphalus *mitochondrial tRNAs follow this pattern. One exception is tRNA(M), which has the anticodon CAT (to recognize both ATG and ATA), as is almost universally the case for all animal mitochondrial systems. In some cases the C is known to be post-transcriptionally modified to 5-formylcytidine to enable the necessary pairing with the ATA codon [32]. However, it is less common that the tRNA(S) expected to recognize codon AGN has a GCT anticodon, since this requires the G to pair with all four nucleotides in the wobble position of AGN codons. It is clear the AGA and AGG codons are being used and are not stop codons (as is the case in vertebrate mtDNAs), since they appear in the reading frames of protein encoding genes 117 times. GCT is used as the tRNA(S) anticodon for all of the cephalopods with complete mtDNA sequences (above), and it is likely that this anticodon is modified post-transcriptionally for all, as is known to occur for the *Loligo bleekeri *tRNA(S), for which the G is modified to 7-methylguanosine [33].

### Non-coding regions

The mtDNA of *N. macromphalus *has 1,416 nucleotides that are not assigned to genes. This is not an unusually large number, but it is atypical that they are distributed among so many regions of the genome (Table 1 and Additional file 3). It is particularly unusual to find this in a mitochondrial genome that has not undergone significant rearrangements, since intergenic non-coding regions appear in some cases to be vestiges of pseudogenes generated by the gene duplication-random loss process of rearrangement [12,16,17,31].

In the largest non-coding region, between *trnQ *and *trnT*, and beginning adjacent to a (CA)_13 _run (see below), there are six repeats of a 62 nucleotide element followed by a partial repeat of 39 nucleotides. Within this are five overlapping regions that have potential for forming tRNA-like structures (Fig. 3). The anticodon portion of these structures is AGT, which would pair with codon ACT (or perhaps ACN) to specify threonine. However, having A in this anticodon position would be very unusual and there is little sequence similarity to *trnT *(or any other tRNA).

Tandem repeats of CA are common, with (CA)_3 _in each of the intergenic regions of *trnA-trnR *and *trnG-atp6 *and an especially noteworthy (CA)_13 _in the region between *trnQ *and *trnT. *Homopolymer runs of T_10_, nine C_9_, and A_20 _are in the regions *trnQ-trnT*, *trnG-atp6*, and *trnE-cox3*, respectively. Non-coding, non-functional portions of mtDNA are generally eliminated rapidly [34], presumably due to selection for small size at the point of entry into the primordial germ plasm during embryogenesis [35], but whether these or any particular motif plays any role in regulating replication of transcription awaits experimentation.

### Base composition and codon usage

The *N. macromphalus *mtDNA is 59.6% A+T. The strand that includes *cox1*, which we will arbitrarily designate as the plus strand for the purpose of discussion, is 33.7% A, 25.8% T, 11.9% G, and 28.5% C. This strand is strongly skewed (as calculated in [20]) away from both T (T-skew = - 0.133) and G (G-skew = - 0.412) in favor of A and C (Table 2). As can be seen in Table 3, this is strongly reflected in the use of synonymous codons. For example, while TTT and TTC are used with approximately equal frequency to specify phenylalanine in plus-strand genes, the bias is 158 to 3 for their usage in minus-strand genes. The use of G vs. A in UUR (leucine) codons is in the ratio of 16 to 89 for plus-strand genes but, even though the mtDNA is A+T-rich, it is 195 to 60 for minus-strand genes. Presumably the biased use of synonymous codons is driven by strand-specific mutational propensity.

**Figure 4:**
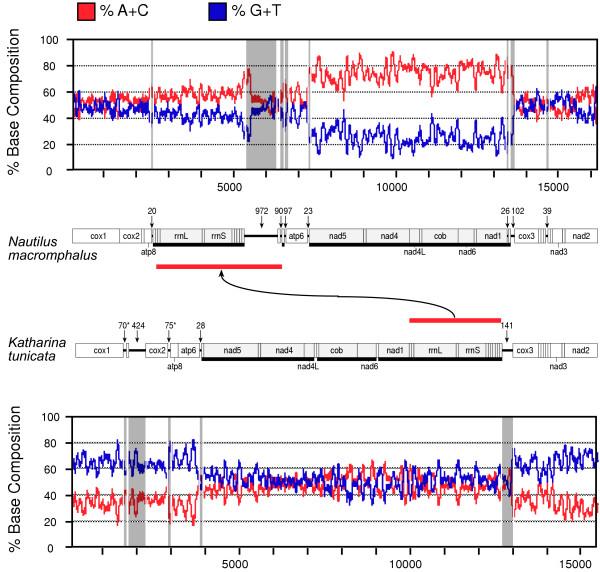
**Plot of A+C and G+T composition along mtDNAs of *Nautilus macromphalus *and *Katharina tunicata *using a sliding window of 100 nucleotides**. In each case, the nucleotide composition is being shown for the strand reported, i.e. the one that is the sense strand for *cox1*. Numbering of nucleotides begins at the arbitrarily chosen *cox1 *(as in Additional file 4 for *N. macromphalus*). The scaled gene maps are also presented. tRNA genes are pictured but not labeled. Underlining and light shading indicates opposite, i.e. right-to-left transcriptional orientation. Numerals label each non-coding region larger than 20 nts, which are then projected onto the plot by gray highlighting. Several of these correspond to positions where there is a shift in nucleotide bias. Asterisks beside two of the numerals for *K. tunicata *indicate some ambiguity where these may instead be supernumerary tRNA genes [28]. Red bars show the major transposition between the two genomes (see Fig. 2).

The minus-strand genes of *N. macromphalus *are organized into three blocks: *trnE *through *nad5*; *trnG *individually; and *trnQ *through *trnF*. As can be seen in Figure 4, each of these is flanked by non-coding regions at least 20 nucleotides in length (Table 1, Additional file 3) and the two largest are delimited by sharp transitions in the ratio of A+C to G+T between the strands, with a strong bias toward A+C in the reported strand for these three regions. That bias is weaker for the region that is predominantly composed of the ribosomal RNA genes, perhaps because of the requirement for base pairing in the secondary structures of the products. There is no significant bias for the plus-strand genes.

The mitochondrial genome of the chiton, *K. tunicata*, contrasts with this. Although the gene arrangement is quite similar, here the pattern of bias is opposite in two different respects. First, it is the plus-strand genes that have strong skew in nucleotide composition, with the minus-strand genes being nearly neutral for this bias. Secondly, the bias for these is strongly toward G+T for the reported strand. Here again, the sharp transitions in base composition are flanked by non-coding regions at least 20 nucleotides in length, which could potentially serve as signaling elements for transcription or replication.

Such skews with one strand being rich in A+C and the other rich in G+T are common for mitochondrial genomes [20]. (See [36] for a review of the proposed causes and an analysis specific to mtDNAs.) This is thought to be due predominantly to the commonality of deamination of adenine and cytosine nucleotides in single-stranded DNA [37-39] which appears transiently during replication and transcription. The relative contribution of these two processes remains unclear [40], as each accounts for one strand being displaced by the nascent DNA or RNA, respectively. (Although this is not without controversy in the case of mitochondrial replication [41-44]). Deaminated adenine forms hypoxanthine, which pairs with cytosine (rather than thymine) and deaminated cytosine forms uracil, which pairs with adenine (rather than guanine). Therefore, the displaced strand, existing in single-stranded form for sometimes protracted periods, tends to become rich in G+T (the analogs of hypoxanthine and uracil) and its complementary strand, therefore, becomes rich in A+C.

Since *N. macromphalus *and *K. tunicata *mtDNAs each have sharp boundaries in base compositional bias that correspond so precisely to shifts in transcriptional orientation, it appears that lesions in the displaced strand during transcription are an important contribution. On the other hand, the contrast in the bias being strong for the minus-strand genes of *N. macromphalus *and for the plus-strand genes of *K. tunicata *shows that some other factor must be at work.

According to the more long-standing and broadly accepted model of mtDNA replication [41] (but see [42-44]), and demonstrated for the few cases where it has been studied, replication of mtDNA is very slow and very asymmetrical, with one strand in single stranded form for a protracted period, so this may be an important factor in strand compositional bias. The nucleotide skew between the two mitochondrial strands is expected to be a combination of various factors, and one could imagine a model whereby a reversal between *N. macromphalus *and *K. tunicata *in which strand is leading during replication could account for their differing skew patterns. If replication in *K. tunicata *mtDNA were to proceed first in the rightward direction according to Figure 4, then the bias introduced during replication would make the reported strand rich in G+T. This would be reinforced by biases introduced during transcription in the regions of the plus-strand genes, causing especially high bias, and countered by the biases introduced during transcription in the regions of the minus-strand genes, causing them to approximately cancel out. If *N. macromphalus *mtDNA replication were to proceed in the opposite direction, right-to-left as in Figure 4, then the effect would be the opposite, with skew generated by mutational bias during replication reinforcing that from transcription of minus-strand genes and opposing that from transcription of plus-strand genes, and accounting for the patterns shown in Figure 4.

It is not clear whether the isolated *trnG *is transcribed individually or is part of the transcription unit that otherwise ends at *nad5*. Separating *trnG *and *nad5 *is a single plus-strand gene, *atp6*, and it is possible that this is transcribed in reverse as part of the larger transcription unit, with this antisense message excised and degraded. When considering only the composition of the third positions of four-fold degenerate codon families, G and T comprise 0.04 and 0.24 of *atp6*, values nearly identical to 0.08 and 0.26 for the other plus-strand genes collectively. However, A and C are 0.24 and 0.48 for *atp6 *vs. 0.41 and 0.24, respectively, for the other plus-strand genes. Perhaps this indicates a modifying force for mutational bias, perhaps the regular reverse transcription of the gene. On the other hand, *trnG *is flanked by large blocks of non-coding sequence which could potentially be signals for initiating and terminating transcription for this individual gene.

Table 2 compares the mtDNA size, base composition, A+T-richness, and strand skews for *K. tunicata *and the eight cephalopod species with complete mtDNA sequences. The other cephalopods all have strand skew measures that are in the same direction, but of lesser magnitude, than *N. macromphalus*, and all of these cephalopods have strand skews in opposite direction from that of the outgroup *K. tunicata*. There also appears to be a trend for larger mtDNAs in the cephalopods and, for the octopus and squid lineages, for greater A+T-richness after the split of that leading to the nautiloids.

### Potential signaling elements

An attempt was made to find potential regulatory sequence elements by comparing all pairs of non-coding regions that are greater than 13 nucleotides in length (Additional file 3) for any blocks 10 nucleotides or longer with identity at least 80% while considering both strands. In addition to the homopolymer runs and dinucleotide repeats discussed above and underlined in Additional file 3, three elements were identified, all associated with reversals in transcriptional orientation. In the largest non-coding region between the oppositely oriented *trnQ *and *trnT *is the sequence TTAAAACAA, also found in the region between *atp6 *and *nad5*. Although both are at a point where transcriptional orientation reverses, the first case is of genes arranged head-to-head but the second of genes tail-to-tail. Also in the *trnQ-trnT *region is the sequence CCNATTTTA which is also found in the region between *trnT *and *trnG*; again in the first case the genes are head-to-head and in the second tail-to-tail. The sequence ATAACAAAACTA occurs in the region between *trnE *and *cox3 *and also between *trnG *and *atp6*, in each case pairs of genes arranged head-to-head. (There are three cases total where genes are arranged head-to-head, these two plus *trnQ-trnT*. There are three cases total where genes are arranged tail-to-tail, *trnT-trnG*, *atp6-nad5*, and *atp8-trnF*.) None of these sequences are present in *K. tunicata *mtDNA and none are present in the non-coding regions of any of the other studied cephalopods.

A comparison was also made between each non-coding region of *N. macromphalus *and each non-coding region of all of the other cephalopod mtDNAs greater than 19 nucleotides in length for any blocks of length 20 or greater matching at least 70%. Although some matches were found, none were consistent across all (or even most) species. Lastly, a search was made for all available cephalopod mtDNAs for long stretches of alternating CA or TA, suggested to play a role in regulation of replication and/or transcription. Of note is that *N. macromphalus *has several regions of alternating CA (Additional file 3), the longest of which is (CA)_13_. Only two of the other cephalopods, *L. bleekeri *and *T. pacificus *have any as long as (CA)_4_. In contrast, while *N. macromphalus *has no regions of alternating TA longer than (TA)_3 _(which occurs in eight places), each of the other cephalopods has many such regions at least of length (TA)_9 _(*T. pacificus *and *W. scintillans*), and some as long as (TA)_22 _(*S. lessoniana*). (The longest alternating TA for *L. bleekeri *is (TA)_14_, for *O. ocellatus *is (TA)_16_, and for both *S. officinalis *and *O. vulgaris *is (TA)_12_.) Of course, it is possible that actual regulatory elements may be more complex and difficult to identify.

## Conclusion

To date, complete mtDNA sequences had been determined for 23 mollusks, a very small sampling compared to those available for vertebrates or arthropods [23]. Even these few studies have revealed that mollusks' mtDNAs have much variation in their features, including losses and gains of genes [2], unusually large amounts of duplicated or non-coding nucleotides [15,24], numerous gene rearrangements [2,25], and doubly uniparental inheritance [26,27]. By contrast, the mtDNA of the cephalopod *Nautilus macromphalus *is fairly typical in many respects, with a size, gene content, and A+T-richness similar to those most common for animal mtDNAs. There have been only a few gene arrangements in this lineage even since its divergence from the basal mollusk group Polyplacophora, and these rearrangements can be confidently polarized among the two lineages by comparing them to mtDNAs of less related animals.

There is strong skew in the distribution of nucleotides between the two strands and it appears that biases in mutational spectrum during both transcription and replication are responsible for this. Compared with most animal mtDNAs, there are a large number of non-coding regions. Although their functions, if any, are not known, the fact that several are at positions of abrupt shift in nucleotide skew and that some contain identical sequence elements suggests that they may contain regulatory signals for transcription and/or replication. This appears to be another example where polyadenylation of tRNAs creates part of the amino-acyl acceptor stem. These, and other features can be interpreted in detail for the systems of these diminutive genomes, and further sampling of complete mtDNA sequences across the tree of life promises to provide insights into general aspects of genome evolution.

## Methods

### Molecular techniques

Testis tissue, stored for longer than a decade at -80°C, but without any record of which species of Nautilus had been sampled, was the gift of Wesley Brown. Fortunately, GenBank contains a short fragment of mitochondrial rRNA for each of the six species of the genus, and this was used for specific identification. The 401 nucleotides in common for this sample and the GenBank records were compared to determine that only two positions differ with the record of *N. macromphalus *(this 0.5% difference is presumably due to intraspecies polymorphism), whereas all others differ by from 16 to 24 positions; therefore, it appears that this sample was from *N. macromphalus*.

Mitochondrial DNA was isolated from approximately 1 g of this tissue by first grinding in liquid nitrogen using a mortar and pestle. This powder was dissolved in 14 ml of homogenization buffer (210 mM mannitol, 70 mM sucrose, 50 mM Tris HCl-pH 75, 3 mM CaCl_2_) and processed using a Tissuemizer T-25 (Tekmar) with three strokes of five seconds each. Membranes were lysed by adding 1/10 volume of 20% SDS and incubating for 20 min at RT. A 1/6 volume of saturated CsCl in water was added and this mixture incubated on ice for 15 min. Debris was pelleted at 17,000 × G for 10 minutes at 4°C. Propidium iodide was added to the collected supernatant to a final concentration of 500 μg/ml and the CsCl concentration was adjusted to a density of 1.57 g/ml. Nuclear and mitochondrial DNA were separated by density gradient centrifugation in a VTi65 rotor at 55,000 × G for 15 hours at 21°C. Although no mitochondrial band was visible in the gradient, the region from about 2–10 mm below the nuclear band was collected using a needle. This was then extracted multiple times with water-saturated butanol to remove the propidium iodide and dialyzed against TE for 24 hours with three buffer changes to remove the CsCl, leaving the sample in a 100 μl volume.

This product was used in PCR as in [45] to amplify first several short fragments of *cox1*, *rrnL*, and *cob *using primers found in [45-47]. The fragment of *cox1 *was cloned into pBluescript (Stratagene) that had been digested with *Eco*RV, T-tailed using Taq polymerase, and gel purified using GeneClean (QBiogene). A successful recombinant clone was selected and DNA prepared using standard techniques. The other fragments were purified by three serial passages through an Ultrafree (NMWL 30,000) spin column (Millipore) and sequenced directly. The sequences of these fragments were determined using an ABI377 automated DNA sequencer with BigDye chemistry (Applied Biosystems) according to supplier's instructions.

Primers were designed to known sequences for use in long PCR [48] with rTth-XL polymerase (Applied Biosystems) according to supplier's instructions, sometimes combined with primers to conserved mtDNA regions. Generously overlapping fragments were amplified from *cox1-nad1 *(using conserved *nad1 *primer CCTGATACTAATTCAGATTCTCCTTC), *nad1-cob*, *cob-rrnL*, and *rrnL – cox1 *(using conserved primer 16SARL [47]), jointly comprising the entire mtDNA. Because there was no information available for the gene arrangement, many combinations of primers were tried, but only these reactions gave bright, singular bands during electrophoretic analysis. Sequence was determined for each as above, then by primer walking through each fragment. To ensure accuracy, all sequence was determined from both strands. Sequencing reads were assembled manually and quality verified by eye using Sequence Navigator (Applied Biosystems).

### Gene annotation and analysis

Genes encoding rRNAs and proteins were identified by matching nucleotide or inferred amino acid sequences to those of *K. tunicata *mtDNA [28] through the use of MacVector (Accelrys). Since it is not possible to precisely determine the ends of rRNA genes by sequence data alone, they were assumed to extend to the boundaries of flanking genes. Each protein gene was inferred to begin at an eligible initiation codon nearest to the beginning of its alignment with homologous genes that does not cause overlap with the preceding gene. In five cases, an abbreviated stop codon was inferred where cleavage of a downstream tRNA from the transcript would leave a partial codon of T or TA, such that subsequent mRNA polyadenylation could generate a TAA stop codon; however, in each of these cases, if the reading frame extended through the first legitimate stop codon there would be only a short overlap with the downstream gene. Genes for tRNAs were identified by eye, generically by their ability to fold into a cloverleaf structure and specifically by anticodon sequence. Subsequent analyses, such as counting anticodon usage, calculating nucleotide frequencies and strand skew values, and identifying repeated elements, were performed using MacVector (Accelrys).

## Abbreviations

*cox1*,*cox2*,*cox3*, cytochrome oxidase subunit I, II, and III protein genes; *cob*, cytochrome b gene;*atp6*,*atp8*, ATP synthase subunit 6 and 8 genes;*nad1*,*nad2*,*nad3*,*nad4*,*nad4L*,*nad5*,*nad6*, NADH dehydrogenase subunit 1–6, 4L genes;*trnA*,*trnC*,*trnD*,*trnE*,*trnF*,*trnG*,*trnH*,*trnI*,*trnK*,*trnL1(nag)*,*trnL2(yaa)*,*trnM*,*trnN*,*trnP*,*trnQ*,*trnR*,*trnS1(nct)*,*trnS2(nga)*,*trnT*,*trnV*,*trnW*,*trnY*, transfer RNA genes designated by the one-letter code for the specified amino acid; in cases where there is more than one tRNA for a particular amino acid, they are differentiated by numeral and with anticodon (which is maximally ambiguous, e.g. "nag" rather than "tag", to allow recognizing homology with those of other organisms).

## Supplementary Material

Additional File 1 Gene arrangements. All available complete gene arrangements for mollusk mtDNA Click here for file

Additional File 4 Gaso *Nm*m. To save space the middle portions of many genes are replaced by a numeral indicating the number of omitted nucleotides. Gene orientation is specified by a dart (>). Stop codons are shown by asterisks whether complete or abbreviated, with a plus symbol indicating an alternative that overlaps the downstream gene. Down-facing arrows mark repeats found in the largest non-coding region. When not conforming to the genetic code, the presumed initiator methionine (M) is in parentheses. Click here for file

Additional File 2 Protein lengths Comparisons of the number of amino acids in the inferred proteins between the mtDNAs of the cephalopod *Nautilus *sp. and the polyplacophoran *Katharina tunicata* Click here for file

Additional File 3 Intergenic regions Summary of the 1416 non-coding nts extracted from the intergenic regions of *Nautilus *sp. mtDNA Click here for file

## Figures and Tables

**Table 1:** Number of nucleotides at gene boundaries. Negative numbers refer to overlapping nucleotides.

**Boundary**	**nts**	**Boundary**	**nts**
*cox1 – cox2*	2	*trnH – nad4*	0
*cox2 – trnD*	0	*nad4 – nad4L*	- 7
*trnD – atp8*	1	*nad4L – trnS2(nga)*	14
*atp8 – trnF*	20	*trnS2(nga) – cob*	0
*trnF – trnL2(yaa)*	0	*cob – nad6*	0
*trnL2(yaa) – trnL1(nag)*	- 1	*nad6 – trnP*	2
*trnL1(nag) – rrnL*	0	*trnP – nad1*	5
*rrnL – trnV*	0	*nad1 – trnE*	26
*trnV – rrnS*	0	*trnE – cox3*	102
*rrnS – trnM*	0	*cox3 – trnK*	0
*trnM – trnC*	3	*trnK – trnA*	- 4
*trnC – trnY*	0	*trnA – trnR*	14
*trnY – trnW*	0	*trnR – trnN*	39
*trnW – trnQ*	- 2	*trnN – trnI*	4
*trnQ – trnT*	972	*trnI – nad3*	0
*trnT – trnG*	90	*nad3 – trnS1(nct)*	0
*trnG – atp6*	97	*trnS1(nct) – nad2*	0
*atp6 – nad5*	23	*nad2 – cox1*	2
*nad5 – trnH*	0		

**Table 2:** Base composition of a chiton and cephalopod mtDNAs. This refers in all cases to the strand that is in the sense orientation for *cox1*

Species	Total nt	T	C	A	G	A+T	G-skew	T-skew
*Katharina tunicata*	15532	5905	1847	4876	2891	69.4%	+0.220	+0.095
*Nautilus macromphalus*	16258	4201	4639	5486	1932	59.6%	-0.412	-0.133
*Sepia officinalis*	16163	5561	2880	6188	1534	72.7%	-0.305	-0.053
*Loligo bleekeri*	17210	5586	3357	6679	1588	71.3%	-0.358	-0.089
*Sepioteuthis lessoniana*	16631	5654	3216	6184	1577	71.2%	-0.342	-0.045
*Todarodes pacificus*	20254	6926	3547	7783	1998	72.6%	-0.279	-0.058
*Watasenia scintillans*	20093	6831	3843	7083	2336	69.2%	-0.244	-0.018
*Octopus ocellatus*	15979	5573	2473	6758	1175	77.2%	-0.356	-0.096
*Octopus vulgaris*	15744	5309	2764	6478	1193	74.9%	-0.397	-0.099

**Table 3:** Codon usage for the 13 mitochondrial proteins of *Nautilus macromphalus*. The total number of codons is 3711. Stop codons were not included in this count. Here the plus strand refers arbitrarily to the one that contains *cox1*.

AA	Codon	All protein encoding genes		Plus strand only	Minus strand only	AA	Codon	All protein encoding genes		Plus strand only	Minus strand only
F	TTT	61 %	225	67	158	I	ATT	38 %	141	83	58
	TTC	18 %	65	62	3		ATC	15 %	57	50	7
L	TTA	40 %	149	89	60	M	ATA	19 %	71	49	22
	TTG	57 %	211	16	195		ATG	25 %	92	21	71
S	TCT	22 %	83	35	48	T	ACT	11 %	41	24	17
	TCC	13 %	50	49	1		ACC	12 %	43	39	4
	TCA	15 %	57	49	8		ACA	12 %	44	40	4
	TCG	04 %	16	3	13		ACG	04 %	14	4	10
Y	TAT	25 %	92	14	78	N	AAT	14 %	53	19	34
	TAC	13 %	49	41	8		AAC	12 %	45	40	5
*	TAA	00 %	0	0	0	K	AAA	10 %	36	24	12
*	TAG	00 %	0	0	0		AAG	11 %	42	10	32
C	TGT	18 %	66	6	60	S	AGT	11 %	39	8	31
	TGC	01 %	5	3	2		AGC	05 %	17	12	5
W	TGA	14 %	52	41	11		AGA	12 %	44	24	20
	TGG	17 %	64	16	48		AGG	20 %	73	4	69
L	CTT	22 %	80	53	27	V	GTT	45 %	168	37	131
	CTC	13 %	48	47	1		GTC	08 %	28	26	2
	CTA	21 %	77	72	5		GTA	16 %	61	36	25
	CTG	08 %	28	13	15		GTG	39 %	145	19	126
P	CCT	14 %	53	22	31	A	GCT	24 %	90	31	59
	CCC	08 %	31	26	5		GCC	09 %	35	29	6
	CCA	09 %	33	31	2		GCA	12 %	45	35	10
	CCG	05 %	19	5	14		GCG	06 %	23	0	23
H	CAT	12 %	44	24	20	D	GAT	12 %	45	18	27
	CAC	10 %	36	29	7		GAC	07 %	25	21	4
Q	CAA	12 %	43	34	9	E	GAA	09 %	35	27	8
	CAG	05 %	20	5	15		GAG	15 %	56	14	42
R	CGT	05 %	17	5	12	G	GGT	17 %	62	19	43
	CGC	02 %	9	8	1		GGC	08 %	29	19	10
	CGA	04 %	16	14	2		GGA	28 %	104	54	50
	CGG	05 %	17	4	13		GGG	41 %	153	19	134
