# Supplementary material for: The complete sequence of the mitochondrial genome of Nautilus macromphalus (Mollusca: Cephalopoda)
Source: BMC Genomics. 2006 Jul 19;7:182. doi: 10.1186/1471-2164-7-182 (PMC1544340; doi:10.1186/1471-2164-7-182)
Supplement: Additional File 1 — Gene arrangements. All available complete gene arrangements for mollusk mtDNA [file 1471-2164-7-182-S1.doc]

**Supplementary Table 1 – All available complete gene arrangements for mollusk mtDNA**

All that are cited as “unpublished” are available in GenBank.

| **Binomen and Accession no.** | **Taxonomy** | **Gene arrangement** | **Reference** |
| --- | --- | --- | --- |
| *Venerupis (Ruditapes) philippinarum*  NC_003354 | Bivalvia; Heteroconchia; Veneridae | cox1, L(nag), nad1, nad2, nad4L, I, cox2, P, cob, rrnL, nad4, H, E, S(nga), atp6, nad3, nad5, Y, M, M, D, V, nad6, K, V, F, W, R, L(yaa), G, Q, N, T, C, A, cox3, rrnS | Okazaki and Ueshima, unpublished^a^. |
| *Inversidens japanensis* (female type)  AB055625 | Bivalvia; Palaeoheterodonta; Unionidae | cox1, cox3, atp6, D, nad4L, nad4, ‑nad6, ‑G, ‑nad1, ‑L(yaa), V, ‑I, ‑C, ‑Q, nad5, ‑P, ‑F, ‑cob, ‑N, ‑L(nag), ‑rrnL, ‑Y, ‑T, ‑K, ‑rrnS, ‑R, ‑W, ‑E, ‑S(nga), ‑A, nad3, ‑M, ‑nad2, ‑S(nct), H, cox2 | Okazaki and Ueshima, unpublished^b^. |
| *Inversidens japanensis* (male type)  AB055624 | Bivalvia; Palaeoheterodonta; Unionidae | cox1, cox3, atp6, ‑D, nad4L, nad4, ‑nad6, ‑G, ‑nad1, ‑L(yaa), V, ‑I, ‑C, ‑Q, nad5, ‑F, ‑cob, ‑P, ‑N, ‑L(nag), ‑rrnL, ‑Y, ‑T, ‑K, ‑rrnS, R, ‑W, ‑M, ‑nad2, ‑E, ‑S, ‑S, ‑A, nad3, cox2, H | Okazaki and Ueshima, unpublished^b^. |
| *Lampsilis ornata*  NC_005335 | Bivalvia; Palaeoheterodonta; Unionidae | ‑cox1, ‑cox2, ‑nad3, ‑H, A, S(nga), E, nad2, M, W, R, rrnS, K, T, Y, rrnL, L(nag), N, P, cob, F, ‑nad5, Q, C, I, V, L(yaa), nad1, G, nad6, ‑nad4, ‑nad4L, ‑atp8, ‑D, ‑atp6, ‑cox3 | Serb and Lydeard, 2003 |
| *Mytilus edulis*  NC_006161 | Bivalvia; Pteriomorphia; Mytilidae | cox1, atp6, T, nad4L, nad5, nad6, F, rrnS, G, N, E, C, I, Q, D, rrnL, Y, cob, cox2, K, M, L(nag), L(yaa), nad1, V, nad4, cox3, S, M, nad2, R, W, A, S, H, P, nad3 | Boore, Medina and Rosenberg, 2004; Hoffmann, Boore and Brown, 1992 |
| *Crassostrea gigas*  NC_001276 | Bivalvia; Pteriomorphia; Ostreidae | cox1, rrnL, cox3, I, T, E, cob, D, cox2, M, L(yaa), P, rrnS, K, C, N, rrnS, Y, atp6, G, V, nad2, R, H, nad4, nad5, nad6, Q, nad3, L(nag), nad1, nad4L, W | Kim, Je and Park, unpublished |
| *Loligo bleekeri*  NC_002507 | Cephalopoda; Coleoidea; Loliginidae | cox1, ‑C, ‑Y, ‑E, N, cox2, ‑M, R, ‑F, ‑nad5, ‑nad4, ‑nad4L, T, ‑L(yaa), ‑G, A, D, atp8, atp6, ‑H, ‑L(nag), cox3, nad3, ‑S(nga), ‑cob, ‑nad6, ‑P, ‑nad1, ‑Q, I, ‑rrnL, ‑V, ‑rrnS, ‑W, K, S(nct), nad2 | Tomita et al., 2002; Sasuga et al., 1999; Tomita, Ueda and Watanabe, 1998 |
| *Sepioteuthis lessoniana*  NC_007894 | Cephalopoda; Coleoidea; Loliginidae | cox1, ‑C, ‑Y, ‑E, N, cox2, ‑M, R, ‑F, ‑nad5, ‑nad4, ‑nad4L, T, ‑L(yaa), ‑G, I, ‑rrnL, ‑V, ‑rrnS, ‑W, A, D, atp8, atp6, ‑H, ‑L(nag), cox3, nad3, ‑S(nga), ‑cob, ‑nad6, ‑P, ‑nad1, ‑Q, K, S(nct), nad2 | Akasaki et al., 2006 |
| *Todarodes pacificus*  NC_006354; AB240153 | Cephalopoda; Coleoidea; Ommastrephidae | cox1, cox2, D, atp8, atp6, ‑F, ‑V, ‑rrnS, ‑C, ‑Q, cox3, K, R, S(nct), nad2, cox1, cox2, D, atp8, atp6, ‑nad5, ‑H, ‑nad4, ‑nad4L, T, ‑S(nga), ‑cob, ‑nad6, ‑P, ‑nad1, ‑L(yaa), ‑L(nag), ‑rrnL, ‑M, ‑Y, ‑W, ‑G, ‑E, cox3, A, N, I, nad3 | Yokobori et al., 2004; Akasaki et al., 2006 |
| *Watasenia scintillans*  NC_007893 | Cephalopoda; Coleoidea; Enoploteuthidae | cox1, cox2, D, atp8, atp6, ‑F, ‑V, ‑rrnS, ‑M, ‑C, ‑Q, cox3, K, R, S(nct), nad2, cox1, cox2, D, atp8, ‑nad5, ‑H, ‑nad4, ‑nad4L, T,‑S(nga), ‑cob, ‑nad6, ‑P, ‑nad1, ‑L(yaa), ‑L(nag), ‑rrnL, ‑Y, ‑W, ‑G, ‑E, cox3, A, N, I, nad3 | Akasaki et al., 2006 |
| *Octopus ocellatus*  AB240156 | Cephalopoda; Coleoidea; Octopodidae | cox1, cox2, D, atp8, atp6, ‑F, ‑nad5, ‑H, ‑nad4, ‑nad4L, T, ‑S(nga), ‑cob, ‑nad6, ‑P, ‑nad1, ‑L(yaa), ‑L(nag), ‑rrnL, ‑V, ‑rrnS, ‑M, ‑C, ‑Y, ‑W, ‑Q, ‑G, ‑E, cox3, K, A, R, N, I, nad3, S(nct), nad2 | Akasaki et al., 2006 |
| *Octopus vulgaris*  NC_006353 | Cephalopoda; Coleoidea; Octopodidae | cox1, cox2, D, atp8, atp6, ‑F, ‑nad5, ‑H, ‑nad4, ‑nad4L, T, ‑S(nga), ‑cob, ‑nad6, ‑P, ‑nad1, ‑L(yaa), ‑L(nag), ‑rrnL, ‑V, ‑rrnS, ‑M, ‑C, ‑Y, ‑W, ‑Q, ‑G, ‑E, cox3, K, A, R, N, I, nad3, S(nct), nad2 | Yokobori et al., 2004 |
| *Sepia officinalis*  NC_007895 | Cephalopoda; Coleoidea; Sepiidae | cox1, cox2, atp8, atp6, ‑F, ‑nad1, ‑L(yaa), ‑L(nag), ‑rrnL, ‑V, ‑rrnS, ‑C, ‑Y, ‑Q, ‑G, N, I, nad3, D, ‑nad5, ‑H, ‑nad4, ‑nad4L, T, ‑S(nga), ‑cob, ‑nad6, ‑P, ‑M, ‑W, ‑E, cox3, K, A, R, S(nct), nad2 | Akasaki et al., 2006 |
| *Aplysia californica*  NC_005827 | Gastropoda; Heterobranchia; Opisthobranchia; Aplysiidae | cox1, V, rrnL, L(nag), A, P, nad6, nad5, nad1, Y, W, nad4L, cob, ‑D, F, cox2, G, H, ‑Q, L(yaa), ‑atp8, ‑N, C, ‑atp6, ‑R, ‑E, ‑rrnS, ‑M, ‑nad3, ‑S(nga), S(nct), nad4, ‑T, ‑cox3, I, nad2, K | Nahir, Kohn and Moroz, unpublished |
| *Pupa strigosa*  NC_002176 | Gastropoda; Heterobranchia; Opisthobranchia; Acteonidae | cox1, V, rrnL, L(nag), A, P, nad6, nad5, nad1, Y, W, nad4L, cob, D, F, cox2, G, H, ‑Q, ‑L(yaa), ‑atp8, ‑N, C, ‑atp6, ‑R, ‑E, ‑rrnS, ‑R, ‑nad3, ‑S(nga), S(nct), nad4, T, ‑cox3, I, nad2, K | Kurabayashi and Ueshima, 2000 |
| *Roboastra europaea*  NC_004321 | Gastropoda; Heterobranchia; Opisthobranchia; Polyceridae | cox1, V, rrnL, L(nag), A, P, nad6, nad5, nad1, Y, W, nad4L, cob, D, F, cox2, G, H, C, ‑Q, ‑L(yaa), ‑atp8, ‑N, ‑atp6, ‑R, ‑E, ‑rrnS, ‑M, ‑nad3, ‑S(nga), S(nct), nad4, ‑T, ‑cox3, I, nad2, K | Grande et al., 2002 |
| *Haliotis rubra*  NC_005940 | Gastropoda; Vetigastropoda; Haliotidae | cox1, cox2, atp8, atp6, ‑F, ‑nad5, ‑H, ‑nad4, ‑nad4L, T, ‑S(nga), ‑cob, ‑nad6, ‑P, ‑nad1, ‑L(yaa), ‑L(nag), ‑rrnL, ‑V, ‑rrnS, ‑M, ‑Y, ‑C, ‑W, ‑Q, ‑G, ‑E, cox3, D, K, A, R, I, nad3, N, S(nct), nad2 | Maynard et al., unpublished |
| *Biomphalaria glabrata*  NC_005439 | Gastropoda; Pulmonata; Planorbidae | cox1, V, rrnL, L(nag), A, P, nad6, nad5, nad1, nad4L, cob, D, C, F, cox2, Y, W, G, H, Q, ‑L(yaa), ‑atp8, ‑N, ‑atp6, ‑R, ‑E, ‑rrnS, M, ‑nad3, ‑S(nga), S(nct), nad4, ‑T, ‑cox3, I, nad2, K | DeJong, Emery and Adema, 2004 |
| *Albinaria coerulea*  NC_001761 | Gastropoda; Pulmonata; Clausiliidae | cox1, V, rrnL, L(nag), P, A, nad6, nad5, nad1, nad4L, cob, D, C, F, cox2, Y, W, G, H, ‑Q, ‑L(yaa), ‑atp8, ‑N, ‑atp6, ‑R, ‑E, ‑rrnS, ‑M, ‑nad3, ‑S(nga), S(nct), nad4, ‑T, ‑cox3, I, nad2, K | Hatzoglou, Rodakis and Lecanidou, 1995 |
| *Euhadra herklotsi*  Z71693‑701  (NOT complete) | Gastropoda; Pulmonata; Bradybaenidae | cox1, V, rrnL, L(nag), P, A, nad6, nad5, nad1, nad4L, cob, D, C, F, cox2, G, H, Y, ‑W, ‑Q, ‑L(yaa), ‑atp8, ‑N, ‑atp6, ‑R, ‑E, ‑rrnS, ‑M, ‑nad3, ‑S(nga), S(nct), nad4, ‑T, ‑cox3, I, nad2, K | Yamazaki et al., 1997 |
| *Cepaea nemoralis*  NC_001816 | Gastropoda; Pulmonata; Helicidae | cox1, V, rrnL, L(nag), A, nad6, P, nad5, nad1, nad4L, cob, D, C, F, cox2, Y, W, G, H, ‑Q, ‑L(yaa), ‑atp8, ‑N, ‑atp6, ‑R, ‑E, ‑rrnS, ‑M, ‑nad3, ‑S(nga), ‑T, ‑cox3, S(nct), nad4, I, nad2, K | Yamazaki et al., 1997; Terrett, Miles and Thomas, 1996 |
| *Katharina tunicata*  NC_001636 | Polyplacophora; Chitonida; Mopaliidae | cox1, D, cox2, atp8, atp6, ‑F, ‑nad5, ‑H, ‑nad4, ‑nad4L, T, ‑S(nga), ‑cob, ‑nad6, P, ‑nad1, ‑L(yaa), ‑L(nag), ‑rrnL, ‑V, ‑rrnS, ‑M, ‑C, ‑Y, ‑W, ‑Q, ‑G, ‑E, cox3, K, A, R, N, I, nad3, S(nct), nad2 | Boore and Brown, 1994 |
| *Graptacme eborea*  NC_006162 | Scaphopoda; Dentaliidae | cox1, S, N, nad2, cob, H, ‑cox2, ‑Q, G, ‑cox3, ‑Y, R, S, ‑nad6, ‑P, ‑nad1, ‑atp8, ‑I, ‑T, rrnS, ‑M, ‑rrnL, V, A, nad3, L(nag), L(yaa), E, W, ‑F, ‑K, ‑nad5, ‑D, ‑nad4, ‑nad4L, atp6, C | Boore, Medina and Rosenberg, 2004 |
| *Siphonodentalium lobatum*  NC_005840 | Scaphopoda; Siphonodentaliidae | cox1, L(yaa), G, ‑T, R, nad2, nad4, I, nad1, nad5, ‑Y, ‑nad4L, ‑atp8, ‑H, ‑A, ‑W, ‑M, ‑V, ‑nad6, ‑Q, ‑K, rrnS, ‑P, ‑N, ‑S(nct), ‑cob, ‑cox2, ‑cox3, ‑C, ‑atp6, ‑S(nga), ‑nad3, ‑E, ‑D, ‑F, ‑rrnL, ‑L(nag) | Dreyer and Steiner, 2004 |

Akasaki T, Nikaido M, Tsuchiya K, Segawa S, Hasegawa M, Okada N: **Extensive mitochondrial gene arrangements in coleoid Cephalopoda and their phylogenetic implications** *Mol Phylogenet Evol* 2006, 38: 648-658

Boore JL, Brown WM: **Complete DNA sequence of the mitochondrial genome of the black chiton, *Katharina tunicata*** *Genetics* 1994, 138 (2): 423-443

Boore JL, Medina M, Rosenberg LA: **Complete sequences of the highly rearranged molluscan mitochondrial genomes of the scaphopod *Graptacme eborea* and the bivalve *Mytilus edulis*** *Mol Biol Evol* 2004, 21(8): 1492-1503

DeJong RJ, Emery AM, Adema CM: **The mitochondrial genome of *Biomphalaria glabrata* (Gastropoda: Basommatophora), intermediate host of *Schistosoma mansoni*** *J Parasitol* 2004, 90(5): 991-997

Dreyer H, Steiner G: **The complete sequence and gene organization of the mitochondrial genome of the gadilid scaphopod *Siphonondentalium lobatum* (Mollusca)** *Mol Phylogenet Evol* 2004, 31(2): 605-617

Grande C, Templado J, Cervera JL, Zardoya R: **The complete mitochondrial genome of the nudibranch *Roboastra europaea* (Mollusca: Gastropoda) supports the monophyly of opisthobranchs** 2002, *Mol Biol Evol* 19(10): 1672-1685

Hatzoglou E, Rodakis GC, Lecanidou R: **Complete sequence and gene organization of the mitochondrial genome of the land snail *Albinaria coerulea*** *Genetics* 1995, 140(4): 1353-1366

Hoffmann RJ, Boore JL, Brown WM: **A novel mitochondrial genome organization for the blue mussel, *Mytilus edulis*** *Genetics* 1992, 131(2): 397-412

Kim S-H, Je E-Y, Park D-W: ***Crassostrea gigas* mitochondrial DNA** Unpublished

Kurabayashi A, Ueshima R: **Complete sequence of the mitochondrial DNA of the primitive opisthobranch gastropod *Pupa strigosa*: systematic implication of the genome organization** *Mol Biol Evol* 2000, 17(2): 266-277

Maynard BT, Kerr LJ, McKiernan JM, Jansen ES, Hanna, PJ: **Mitochondrial DNA sequence and gene organization of the Australian blacklip abalone, *Haliotis rubra* (Leach)** Unpublished

Nahir B, Kohn AB, Moroz LL: **Complete DNA sequence of the mitochondrial genome of the marine opisthobranch mollusc, *Aplysia californica*** Unpublished

Okazaki M, Ueshima R: **Gender-associated mtDNA of *Tapes philippinarum*** Unpublished^a^

Okazaki M, Ueshima R: **Evolutionary diversity between the gender-associate mitochondrial DNA genomes of freshwater mussels** Unpublished^b^

Sasuga J, Yokobori S–I, Kaifu M, Ueda T, Nishikawa K, Watanabe K: **Gene contents and organization of a mitochondrial DNA segment of the squid *Loligo bleekeri*** *J Mol Evol* 1999, 48(6): 692-702

Serb JM, Lydeard C: **Complete mtDNA sequence of the North American freshwater mussel, *Lampsilis ornata* (Unionidae): An examination of the evolution and phylogenetic utility of mitochondrial genome organization in Bivalvia (Mollusca)** *Mol Biol Evol* 2003, 20(11): 1854-1866

Terrett JA, Miles S, Thomas RH: **Complete DNA sequence of the mitochondrial genome of *Cepaea nemoralis* (Gastropoda: Pulmonata)** *J Mol Evol* 1996, 42(2): 160-168

Tomita K, Ueda T, Watanabe K: **7-Methylguanosine at the anticodon wobble position of squid mitochondrial tRNA(Ser)GCU: molecular basis for assignment of AGA/AGG codons as serine in invertebrate mitochondria** *Biochim Biophys Acta* 1998, 1399(1): 78-82

Tomita K, Yokobori S–I, Oshima T, Ueda T, Watanabe K: **The cephalopod *Loligo bleekeri* mitochondrial genome: Multiplied noncoding regions and transposition of tRNA Genes** *J Mol Evol* 2002, 54(4): 486-500

Yamazaki N, Ueshima R, Terrett JA, Yokobori S–I, Kaifu M, Segawa R, Kobayashi T, Numachi K, Ueda T, Nishikawa K, Watanabe K, Thomas RH: **Evolution of pulmonate gastropod mitochondrial genomes: comparisons of complete gene organization of *Euhadra*, *Cepaea* and *Albinaria* and implications of unusual tRNA secondary structure** *Genetics* 1997, 145(3): 749-58

Yokobori S–I, Fukuda N, Nakamura M, Aoyama T, Oshima T: **Long-term conservation of six duplicated structural genes in cephalopod mitochondrial genomes** *Mol Biol Evol* 2004, 21(11): 2034-2046
