# Supplementary material for: The complete sequence of the mitochondrial genome of Nautilus macromphalus (Mollusca: Cephalopoda)
Source: BMC Genomics. 2006 Jul 19;7:182. doi: 10.1186/1471-2164-7-182 (PMC1544340; doi:10.1186/1471-2164-7-182)
Supplement: Additional File 4 — Gaso Nmm. To save space the middle portions of many genes are replaced by a numeral indicating the number of omitted nucleotides. Gene orientation is specified by a dart (>). Stop codons are shown by asterisks whether complete or abbreviated, with a plus symbol indicating an alternative that overlaps the downstream gene. Down-facing arrows mark repeats found in the largest non-coding region. When not conforming to the genetic code, the presumed initiator methionine (M) is in parentheses. [file 1471-2164-7-182-S4.doc]

10 20 1520 1530 1540 1550 2200

ATGCGATGAGTATTTTCTACA-/1489/-AAACAGGAGCTCTTACATTGTAGAAATGGCCTTATGAGGACAAATTAA-/632/-TCTTATTGTGGTTATC

M R W V F S T E T G A L T L *** M A L W G Q I K F L L W L S

_______________________*cox1*________________________> ________________________*cox2*__________________

2210 2220 2230 2240 2250 2260 2270 2280 2290 2300

AAAAAACATTTAAAAAATGAGTTATGTATTAACGTAGGACCGTCACTCCTAAGTCATCACAGAAGTGATATTTTTTAGATGCCTCAACTATCACCCCTCA

K N I *++ M P Q L S P L

__*cox2*____>______________________________*trnD*_______________________________> _______*atp8*___________

2430 2440 2450 2460 2470 2480 2490 2500 2510

-/114/-CTCCCCACTATAAGTGATGATAACCTAACTACTATTTTCATTATACATATTAGTTTACATCAACCTCCTCAGCACCTTCAACGCTGCGCTCTT

P P H Y K W W *** <_________________________*trnF*____________________

__*atp8*_______________________>

2520 2530 2540 2550 2560 2570 2580 2590 2600 2610

ATAAGCTAAATAAGTTATTAAGAAAGCTCACTACTTATTCCTAGAGCTTAAATCTATCGTACTATTCTACCACCTTAATTTTGGGTAGGTTAACTTAATC

______*trnF*_____ <_______*trnL1*(tag)____

<_________________________*trnL2*(taa)____________________________

2610 2620 2630 2640 2650 2660 2670 2680 2690 2700

GGTTAACTTAATCCTATCCATCGATCCTAAATCGATTACACTCATCTGCCAACCCAAATCAAGAAACTTAAATTTAAATTTTAAACACACATTTAATTCT

_______________________*trnL1*(tag)_________________________<_________________*rrnL*____________________

3990 4000 4010 4020 4030 4040 4050 4060 4070

-/1280/-TATTCACTCACATAAGACTACCCGCTGTGAAAAGAGTAATATTTACTATTAACCAAGCGTAAAAAGGTCACATTTACTTATGTTACTTCTCA

__*rrnL*____________________________<___________________________________*trnV*__________________________

4080 4090 4100 4950 4960 4970 4980 4990 5000 5010

CTTCCAGAAGCAGGTTCCCCTACCTCTA-/845/-CAACCAGAACCAAATTGTTAGCAAGAAATTGAAACAAATTATTTTTGGGGTATGAACCCACTAGC

_____________________________*rrnS*___________________><______________________*trnM*____________________

5020 5030 5040 5050 5060 5070 5080 5090 5100 5110

TTACATTAGCTTATCTTACTACTAAGCCTACACCACTAAGTACCTATGAACTTGCAATTCATTGTTCTATTCAAACTCCTAAGCCTTATGAGAGGGCTTA

__*trnM*______________ <___________________________*trnC*______________________________<_____*trnY*_____

5120 5130 5140 5150 5160 5170 5180 5190 5200 5210

TACACCCATTAAATGAATCTACAATTCAACACCTAAAAATCAGCCACCTCACACAAGACCTACGCTTTCGACATATCATAAGTTTTGAAGACTAATAGTT

___________________*trnY*______________________________<____________________*trnW*______________________

5220 5230 5240 5250 5260 5270 5280 5290 5300 5310

TACATTAACCTAAGACCTTGTAGGAGAGGGTCCGAACCCTCACTTAAAAACCCAAATCTTCTCGTGCACTCCACACCACCCCACATAAACTAACAACCTG

__*trnW*_____________

<______________________________*trnQ*_________________________________

5320 5330 5340 5350 5360 5370 5380 5390 5400 5410

CTCTATTAAAACAAGTAAAGATGACCCCAACAACTAACAACTTACACAAAGTTTACGTTCTACCTTGACACCCACACTTTTAACATATACCTAAACATGG

5420 5430 5440 5450 5460 5470 5480 5490 ↓ 5500 5510

TACCAACCGATAATCACCTTATACCCCCTTACTTCCCCACACACCTAACACACACACACACACACACACACACAGAAGTTAAAGTACTAACCGGTAATCA

5520 5530 5540 5550 ↓5560 5570 5580 5590 5600 5610

CTCTATACACTGTTTACTCATACTACTAACATATACTAGGTTAGGGTACTAACCGGTAATCACTCTATACACTGTTTACTCATACTACTAACATATACTA

↓ 5630 5640 5650 5660 5670 ↓ 5690 5700 5710

GGTTAGGGTACTAACCGGTAATCACTCTATACACTGTTTACTCATACTACTAACATATACTAGGTTAGGGTACTAACCGGTAATCACTCTATACACTGTT

5720 5730 5740 ↓ 5750 5760 5770 5780 5790 5800 ↓ 5810

TACTCATACTACTAACATATACTAGGTTAGGGTACTAACCGGTAATCACTCTATACACTGTTTACTCATACTACTAACATATACTAGGTTAGGGTACTAA

5820 5830 5840 5850 5860 ↓5870 5880 5890 5900 5910

CCGGTAATCACTCTATACACTGTTTACTCATACTATTAACATATACTAGGTTAGGGTACTAACCGGTAATCACTCTACACACTGTTTACTTATTCTTATT

5920 5930 5940 5950 5960 5970 5980 5990 6000 6010

ACTCATATGGACATAATCTATACATCTTGTTCTATACACATGTGTTCCACCCTATATACTGTCTATATACCCACTCTATACACCTTTCTTTCATTCATTC

6020 6030 6040 6050 6060 6070 6080 6090 6100 6110

ATTCATATCTATATTCCTTCTTATTCTATATTCTATTCCTATTCTTCCATTTCTCGCATCCTATATACATCTAGCCCAATGTGGGCTATGCGCGAAAGTT

6120 6130 6140 6150 6160 6170 6180 6190 6200 6210

GTTTTTATAACTTTTTCATAGAAAATCGGCCCTTTTTTTTTTCAGTGCCTATTTTGAACTGTAATGCAATCACCTCAAAACAGGGCTAAATAAAATATTT

6220 6230 6240 6250 6260 6270 6280 6290 6300 6310

ATAAACATTACCCCTATGTGGTCAAAATCCCCCAATTTTAAGGAGTTTCCCGTAGCAGCCCTGGAAGCTTGTTTTAAGTAGCGGCCTTGTAAACCGAAGA

___________________*trnT*____________________

6320 6330 6340 6350 6360 6370 6380 6390 6400 6410

TTGTGATACTAAATCTCTCAGGGCAGTAAATTTTTCGTAATTTCCATCATCTGTTTCGCTTAAACGCACCTATTTTAAAGTCATTCTCTAGATATTTGGG

_____*trnT*_______________>

6420 6430 6440 6450 6460 6470 6480 6490 6500 6510

CCCCCCATACCCTTATATCTACTCTAAACGATTTTAGACCTAATGCTTGGAAGGCACCTATACTTATTATACTAAAAAGACATCCTCACCCCACCTGGAT

<_____________________________*trnG*________________________________

6520 6530 6540 6550 6560 6570 6580 6590 6600 6610

TCGCTGTGGGCTTATCTTTGTCCCCCCCCCTTCCTTCAGCAGCTCAATAACAAAACTACAAAACAACCCTCCACACATATGCTATCTGACATCTTCTCAA

M L S D I F S

____________*atp6*______

7270 7280 7290 7300 7310 7320 7330 9000

-/650/-TATTCAGACGATCACGCTAATTAGATAACCTTACCTTTAAAACAATTCTAAATAAAAACAACACCAACCCCTAC-/1653/-TACTAGCCTTC

Y S D D H A N *** *** I F V V G V G V S A K

__*atp6*________________________> <____________________________*nad5*_____________

9010 9020 9030 9040 9050 9060 9070 9080 9090 9100

AAATAAAACTTCAAATCAACAAACACTATGAAATGGTTACCCCTTCGCCAACACCACAACTTGACATTTTACACATAAACTAATTCCACTAATACCACCA

L Y F K L D V F (M)<_____________________________*trnH*____________________________+** Y W W

__________*nad5*____________ <____*nad4*__

9110 10420 10430 10440 10450 10460 10710 10720

ATACAGTCCC-/1300/-CCCCTAACGCAAAAACACAACCTAACACTTATACAACCTCAAACCCCTTA-/240/-AAACCAGCGAATATAACAAAACCAT

Y L G G L A F V C G L (M)

___________*nad4*_______________________________

*** C K Y L S L G S V V L S Y L L V M

<_______________________________*nad4L*________________________

10730 10740 10750 10760 10770 10780 10790 10800 10810 10820

TTCTAACTATGCTAAACCATTAAGCGACTCGAACACCTCCACATCTGCTTTCAAAACAAACCCTAACCTTTAGTAATGGCTACCCCAACAAAACAACACT

<____________________________*trnS2(tga)*__________________________+** G L L V V S

<_________*cob*_______

10830 11920 11930 11940 11950 119600 12430 12440

AATATACCC-/1077/-ATGTCTTTTTCGAATAGACCTAAGCATAATACATACTAAAAGGCCGCAGAGG-/456/-ACAAAACACCACTATTAATCTCAT

I Y G H S K R I S S L M

___________*cob*______________________________ Y M S F P R L P C F V V M L S M

+++ <__________________________*nad6*_________________________

12450 12460 12470 12480 12490 12500 12510 12520 12530 12540

ATTCAGAAGATAAGGCCTTCCTCAATCACTAATTTCCAATATTAAAATTTTACATAAACTACCTTCTGCACCCTTAGCCCCACCCTATAACCCTCACCCT

<____________________________*trnP*_________________________________ *** G W G M V S V S

<____________*nad1*__________

13430 13440 13450 13460 13470 13480 13490 13500 13510

-/877/-GTAACCAACACACACAAAACTCTAACCATTACGCTACCCCCACCAAATAAACCCTCTAACCTAGGTGTCACATCACACTCCCTTAAACTGAAA

T V L V C L V S V M

__*nad1*______________________________ <__________________*trnE*______________

13520 13530 13540 13550 13560 13570 13580 13590 13600 13610

CTTTAACGTGCAAACCTATACACCACTAAGCCCCAATCCCACCCCATAAGCTTTCAACAGCTAATTTTCCTCCCACCCAAAAAAAAAAAAAAAAAAAACA

_______*trnE*_____________________

13620 13630 13640 13650 13660 13670 14410 14420

TAACAAAACTATAAATCTATAAACTATTCTCTCAATGATCCGAAATCCCTTTCATTTAGTGGA-/721/-TACACATGTGTATATTGATGAGGATCCTAA

M I R N P F H L V E Y T C V Y W W G S ***

_______________________________*cox3*______________________________>

14440 14450 14460 14470 14480 14490 14500 14510 14520

TTTAAGGTAACTTAACTTATAGTGTTAAACTTTTAATTTAAAAATGGTCGTACAACCCCTTGGCTTTATACTTTAAGAGCTAGAAGCCCTGATTTGCATT

______________________________*trnK*______________________________>

____________________*trnA*_______________

14540 14550 14560 14570 14580 14590 14600 14610 14620

TAGGTAGTAGGATCACCCTTAAAGCCCTTTACACACAAGGGAGAATTAAAGTGAAGTATCACATGCGGTTTCGGCCCGCAAGTTGGAAACCTATTCCTAA

__________*trnA*____________> _____________________________*trnR*__________________________

14640 14650 14660 14670 14680 14690 14700 14710 14720

TTCCTTTAGCCCATTCTTTTACTTCACCTCGCTTTCCTCCTCCCCGAAGAGAAGCTAATAATAGCATTTAATTGTTAATTAAAAAATAGTAATGCATTTT

____> _________________________*trnN*___________________________

14740 14750 14760 14770 14780 14790 14800 14810 14820

ACCATTCGGGCTTGATATTGAGCCGGAATAACGGATTACATTGATGTTGTAAATCACGGACATATTATGTACCCAATATCCGTGTCAATATTAATAGTTG

__*trnN*__> (M) S M L M V

________________________________*trnI*_______________________________>_________*nad3*______

14840 15130 15140 15150 15160 15170 15180 15190 15200

TTTCCTCTATCCT-/280/-CTCCATGAATGGTCTCAAGGGTCCCTTGAGTGAGTCTCCTAAGAATGATTGAGAATGAAGTGGGGCTGCTAACCTTACTT

V S S I L L H E W S Q G S L E W V S ***__________________*trnS1(gct)*__________

_______________*nad3*__________________________________________>

15210 15220 15230 15240 15250 15260 16230 16240 16250

CGGATGGTTCAAAACCATCCCTTCTTTATGTATACTAAAAGATTCCCATTTACGTTCCTA-/960/-AGGTATTGCTATTCTTACTTTTGTGGTCTAATT

________*trnS1(gct)*_________ M Y T K S F P F T F L G I A I L T F V V ***

___________________________________*nad2*________________________________
