# Supplementary material for: The complete sequence of the mitochondrial genome of Nautilus macromphalus (Mollusca: Cephalopoda)
Source: BMC Genomics. 2006 Jul 19;7:182. doi: 10.1186/1471-2164-7-182 (PMC1544340; doi:10.1186/1471-2164-7-182)
Supplement: Additional File 2 — Protein lengths Comparisons of the number of amino acids in the inferred proteins between the mtDNAs of the cephalopod Nautilus sp. and the polyplacophoran Katharina tunicata [file 1471-2164-7-182-S2.doc]

**Supplementary Table 2 – Comparisons of the number of amino acids in the inferred proteins between the mtDNAs of the cephalopod *Nautilus* sp. and the polyplacophoran *Katharina tunicata***

| Protein | *Nautilus* sp. | *K. tunicata* |
| --- | --- | --- |
| Atp6 | 231 | 230 |
| Atp8 | 52 | 53 |
| Cox1 | 510 | 513 |
| Cox2 | 227 | 229 |
| Cox3 | 259 | 259 |
| Cob | 377 | 379 |
| Nad1 | 310 | 316 |
| Nad2 | 342 | 338 |
| Nad3 | 117 | 120 |
| Nad4 | 449 | 442 |
| Nad4L | 97 | 100 |
| Nad5 | 572 | 571 |
| Nad6 | 168 | 166 |
