# Supplementary material for: The complete sequence of the mitochondrial genome of Nautilus macromphalus (Mollusca: Cephalopoda)
Source: BMC Genomics. 2006 Jul 19;7:182. doi: 10.1186/1471-2164-7-182 (PMC1544340; doi:10.1186/1471-2164-7-182)
Supplement: Additional File 3 — Intergenic regions Summary of the 1416 non-coding nts extracted from the intergenic regions of Nautilus sp. mtDNA [file 1471-2164-7-182-S3.doc]

**Supplementary table 1 - Summary of the 1416 non-coding nts extracted from the intergenic regions of *Nautilus* sp. mtDNA.**

Dinucleotide repeats and hompolymer runs pointed out in the text are underlined.

| Region after: | But before: | No. nts | Sequence |
| --- | --- | --- | --- |
| *cox1* | *cox2* | 2 | AA |
| *trnD* | *atp8* | 1 | G |
| *atp8* | *trnF* | 20 | CCTAACTACTATTTTCATTA |
| *trnM* | *trnC* | 3 | ACT |
| *trnQ* | *trnT* | 972 | TAAACTAACAACCTGCTCTATTAAAACAAGTAAAGATGACCCCAACAACTAACAACTTACACAAAGTTTACGTTCTACCTTGACACCCACACTTTTAACATATACCTAAACATGGTACCAACCGATAATCACCTTATACCCCCTTACTTCCCCACACACCTAACACACACACACACACACACACACACAGAAGTTAAAGTACTAACCGGTAATCACTCTATACACTGTTTACTCATACTACTAACATATACTAGGTTAGGGTACTAACCGGTAATCACTCTATACACTGTTTACTCATACTACTAACATATACTAGGTTAGGGTACTAACCGGTAATCACTCTATACACTGTTTACTCATACTACTAACATATACTAGGTTAGGGTACTAACCGGTAATCACTCTATACACTGTTTACTCATACTACTAACATATACTAGGTTAGGGTACTAACCGGTAATCACTCTATACACTGTTTACTCATACTACTAACATATACTAGGTTAGGGTACTAACCGGTAATCACTCTATACACTGTTTACTCATACTATTAACATATACTAGGTTAGGGTACTAACCGGTAATCACTCTACACACTGTTTACTTATTCTTATTACTCATATGGACATAATCTATACATCTTGTTCTATACACATGTGTTCCACCCTATATACTGTCTATATACCCACTCTATACACCTTTCTTTCATTCATTCATTCATATCTATATTCCTTCTTATTCTATATTCTATTCCTATTCTTCCATTTCTCGCATCCTATATACATCTAGCCCAATGTGGGCTATGCGCGAAAGTTGTTTTTATAACTTTTTCATAGAAAATCGGCCCTTTTTTTTTTCAGTGCCTATTTTGAACTGTAATGCAATCACCTCAAAACAGGGCTAAATAAAATATTTATAAACATTACCCCTATGTGGTCAAAATCCCCCAATTTTAAGGAGTTTCCCGTAGCA |
| *trnT* | *trnG* | 90 | GTAAATTTTTCGTAATTTCCATCATCTGTTTCGCTTAAACGCACCTATTTTAAAGTCATTCTCTAGATATTTGGGCCCCCCATACCCTTA |
| *trnG* | *atp6* | 97 | ATCCTCACCCCACCTGGATTCGCTGTGGGCTTATCTTTGTCCCCCCCCCTTCCTTCAGCAGCTCAATAACAAAACTACAAAACAACCCTCCACACAT |
| *atp6* | *nad5* | 23 | ATAACCTTACCTTTAAAACAATT |
| *nad4L* | *trnS2* | 14 | TTCTAACTATGCTA |
| *nad6* | *trnP* | 2 | AT |
| *trnP* | *nad1* | 5 | CACCC |
| *nad1* | *trnE* | 26 | TACGCTACCCCCACCAAATAAACCCT |
| *trnE* | *cox3* | 102 | CCAATCCCACCCCATAAGCTTTCAACAGCTAATTTTCCTCCCACCCAAAAAAAAAAAAAAAAAAAACATAACAAAACTATAAATCTATAAACTATTCTCTCA |
| *trnA* | *trnR* | 14 | TTTACACACAAGGG |
| *trnR* | *trnN* | 39 | TTAGCCCATTCTTTTACTTCACCTCGCTTTCCTCCTCCC |
| *trnN* | *trnI* | 4 | GCTT |
| *nad2* | *cox1* | 2 | TT |
